# Cerebral glucose metabolism in bipolar disorder: A voxel‐based meta‐analysis of positron emission tomography studies

**DOI:** 10.1002/brb3.2117

**Published:** 2021-03-26

**Authors:** Chujun Wu, Chutong Ren, Ziwei Teng, Sujuan Li, Floyd Silva, Haishan Wu, Jindong Chen

**Affiliations:** ^1^ National Clinical Research Center for Mental Disorders Department of Psychaitry China National Technology Institute on Mental Disorders The Second Xiangya Hospital of Central South University Changsha China; ^2^ The Second Xiangya Hospital of Central South University Changsha China; ^3^ University of New Mexico School of Medicine Albuquerque NM USA

**Keywords:** bipolar disorder, cerebral glucose metabolism, PET

## Abstract

**Background:**

Previous positron emission tomography studies have reported the changes of cerebral glucose metabolism in bipolar disorder. However, the findings across studies remain controversial, containing differing results.

**Methods:**

A systematic literature search of the PubMed, Embase, Cochrane Library, and Web of Science databases was conducted. We conducted a voxel‐wide meta‐analysis of cerebral glucose metabolism studies, using the seed‐based mapping approach, in patients with bipolar disorder (BD).

**Results:**

We identified 7 studies suitable for inclusion, which included a total of 126 individuals with BD and 160 healthy controls. The most consistent and robust findings were an increase in cerebral glucose metabolism in the right precentral gyrus and a decrease in the left superior temporal gyrus, left middle temporal gyrus, and cerebellum. Additionally, the sex distribution and illness duration had significant moderating effects on cerebral glucose metabolism alterations.

**Conclusions:**

Cerebral glucose metabolism alterations in these brain regions are likely to reflect the disease‐related functional abnormalities such as emotion and cognition. These findings contribute to a better understanding of the neurobiological underpinnings of bipolar disorder.

Limitations.

This study was done at a study level and cannot be addressed at the patient level. Subgroup analysis of BD I and BD II is not possible due to limited literature data.

## Graphical abstract

The changes of cerebral glucose metabolism in bipolar disorder remains differences. Our meta‐analysis used the seed‐based mapping approach and found that cerebral glucose metabolism alterations are likely to reflect the disease‐related functional abnormalities.

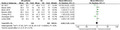

## 1 INTRODUCTION

Bipolar disorder (BD) is a chronic mental disorder, characterized by severe recurring mood swings which cause a reduction in the functioning and quality of life. Bipolar disorder can be classified along a spectrum defined by the severity of mood elevation, from bipolar I to bipolar II (Grande et al., 2016; Kessler et al., 2005). Compared with BD I, the depressive phase of BD II is more severe, while the manic phase mainly manifests as hypomanic state (Tondo et al., 2017). In 2019, the results of a mental health survey in China showed that the lifetime prevalence of bipolar disorder was 0.6% (Huang et al., 2019). Bipolar disorder frequently begins with depressive episodes, easily misdiagnosed as depression, which increases the difficulty in the management of the disease (Murray et al., 2012). Therefore, improving the informed and accurate diagnosis of BD is significantly important in managing the disease.

Brain functional imaging refers to the process of using various imaging techniques to characterize the functional activities of the brain. The application of neuroimaging technology is considered as a possible method for diagnosing mental illnesses as it can contribute to the understanding of the neurobiology and to the development of pathophysiologic models (Bhardwaj et al., 2010). Neuroimaging technology currently being implemented in the diagnosis of mental illnesses includes magnetic resonance imaging (MRI), positron emission tomography (PET), electroencephalography (EEG), and magnetoencephalography (MEG) (Roalf & Gur, 2017). Positron emission tomography (PET), as opposed to other methods, can create functional connectivity maps of distinct spatial distributions of brain regions and measure cerebral glucose metabolism in vivo (Patlak et al., 1983), and is therefore the focused imaging modality of our study. Synthesized first in 1976, 2‐deoxy‐2‐[18F] fluoro‐D‐glucose (FDG) has become the most widely available tracer in the utilization of PET (Ido et al., 1978) and is now being utilized in conjunction with PET to further study patients with mental disorders (Davison & O'Brien, 2014). The measurement of cerebral glucose metabolism using FDG‐PET can be used to assess changes in thinking activities and disease states under noninvasive conditions (Mergenthaler et al., 2013; Scholl et al., 2014). In major depressive disorder patients, the uptake of FDG is reduced in the bilateral insula, right cingulate gyrus, caudate, left putamen, and extranuclear, indicating reduced metabolism in these areas; however, FDG uptake is increased in the bilateral thalamus, right anterior gyrus, and left anterior gyrus, suggesting increased metabolism (Aihara et al., 2007; Brody et al., 2001; Kegeles et al., 2003; Kennedy et al., 2001; Saxena et al., 2001).

In BD patients, the changes of cerebral glucose metabolism were mostly found in the basal ganglia, limbic system, cerebellum, anterior cingulate cortex, DLPFC, and medial temporal structures, but there is no consensus on the results (Brooks & Vizueta, 2014; Maggioni et al., 2017). A review written by Brook et al (Brooks & Vizueta, 2014) reported that the metabolism of depressed BD patients is altered in the amygdala and dorsolateral prefrontal cortex (DLPFC), while the metabolism of manic BD patients seemed to be altered in the anterior cingulate cortex, DLPFC, and medial temporal structures. Therefore, the aim of our meta‐analysis is to elucidate the differences of cerebral glucose metabolism between BD patients and healthy subjects. Finally, we aim to develop an understanding of the pathophysiology of BD in the context of its link with cerebral glucose metabolism.

## 2 METHODS

### 2.1 Literature search and inclusion criteria

This systematic review and meta‐analysis were conducted according to the Preferred Reporting Items for Systematic reviews and Meta‐Analyses (PRISMA) statement (Liberati et al., 2009). Two of the authors (WCJ and RCT) independently searched on PubMed, Web of Science, Embase, and the Cochrane Library up to January 2019, using the following searching terms “(bipolar disorder [Title/Abstract]) AND glucose metabolism [Title/Abstract]”. No language restrictions were applied. After that, an additional search was made in PubMed using “(((((bipolar disorder[Title/Abstract]) AND 18 F‐Fluorodeoxyglucose[Title/Abstract])) OR ((bipolar disorder[Title/Abstract]) AND FDG[Title/Abstract])) OR ((bipolar disorder[Title/Abstract]) AND positron emission tomography[Title/Abstract])) OR ((bipolar disorder[Title/Abstract]) AND PET[Title/Abstract])”. Finally, reference lists of reviews published on similar themes were checked. After a screening based on titles and abstracts, full texts were read intensively to judge whether the article should be included according to the inclusion criteria. A third researcher (TZW) was consulted for resolution when disagreements occurred.

All studies had to meet the following criteria: (a) BD patients fulfilled the diagnostic criteria of the Diagnostic and Statistical Manual of Mental Disorders, 4th edition (DSM‐IV) or the Diagnostic and Statistical Manual of Mental Disorders, 5th edition (DSM‐V); (b) the research design is reasonable and the statistical analysis method is correct according to the Newcastle–Ottawa Scale (NOS), and the experiment involves patients with both bipolar disorder (BD) and healthy controls (HC); (c) the BD groups and HC groups are age and sex matched; (d) cerebral glucose metabolism was measured using FDG‐PET; (e) results were presented in Talairach space or Montreal Neurological Institute (MNI) coordinates; and (f) there were at least 10 subjects in each group.

We excluded studies by the following criteria: (a) studies with incomplete data, such as dissertations and conference abstracts; (b) studies reporting only patients but not healthy controls; (c) studies using regions of interest (ROIs) analysis; and (d) if data from the same patients had been published in multiple literatures, we only retained the literature with the most exhaustive information to avoid duplicate publication bias.

### 2.2 Study quality assessment

In order to exclude possible information bias, a basic appraisal of study quality was assessed using the NOS. Two researchers (WCJ and RCT) independently conducted the quality assessments, and the final score of each study was averaged. Good quality was defined by achieving a minimum score of 7 on this scale. The results of study quality assessment are shown in Table 1.

**TABLE 1:** Clinical characteristics and quality of the 7 studies included in the meta‐analysis

Study	Type of BD	BD patients (female)	HC (female)	Mean Age (*SD*)(years)	Duration (*SD*)(years)	Threshold	Quality
Bauer (2005)	BD I (9)+BD II (1)	10 (10)	10 (10)	39.3 (7.8)	20.4 (7.0)	Uncorrected	★★★★★★★★★
Linda Mah (2006)	BD II	13 (11)	18 (13)	43.0 (8.4)	22.9 (12.0)	Uncorrected	★★★★★★★★
Brooks (2008)	BD I (6)+BD II (9)	15 (8)	19 (7)	36.1 (10.4)	NA	Uncorrected	★★★★★★★★★
Cheng (2012)	BD I (17)+BD II (17)	34 (22)	17 (12)	43.9 (11.9)	13.5 (11.9)	SVC	★★★★★★★★
A C Altamura (2017)	BD I	17 (13)	27 (13)	38.7 (8.2)	11.4 (7.0)	FWE‐corrected	★★★★★★★★
Boen (2018)	BD II	22 (17)	21 (14)	32.6 (6.0)	NA	FWE‐corrected	★★★★★★★★★
Giuseppe Delvecchio (2019)	unclear	15 (8)	48 (33)	59.8 (7.1)	17.7 (9.7)	FWE‐corrected	★★★★★★★★

Abbreviations: BD, bipolar disorder; FWE‐corrected, peak family‐wise error correction; HC, healthy controls; SVC, small‐volume correction.

### 2.3 Meta‐analysis methods

Meta‐analyses were performed using the Review Manager 5.3 (Revman 5.3) and the Seed‐Based Mapping (SDM) software package (www.sdmproject.com).

Revman was used to analyze general demographic information between the groups and show the results by stem‐and‐leaf plot.

SDM uses a voxel‐based meta‐analytic procedure that is improved compared to other existing methods (Radua et al., 2012). SDM used the reported peak coordinates and effect sizes to recreate new brain maps based on the spatial correlation between neighboring voxel and accounts for sample size and variance as well as between‐study heterogeneity (Lim et al., 2014; Radua, 2014).

The SDM methods have previously been described in detail (Radua, 2014). Here is a brief summary. First, the peak coordinates and effect values (such as t value) of BD patients which were different from healthy controls were extracted from each data set. Second, we recreated a standard Montreal Neurological Institute map for each study by means of an anisotropic Gaussian kernel. By doing this, the voxels that are more strongly correlated with peaks are assigned higher effect sizes. Third, a map of the effect size variance was derived for each study from its effect values and sample size. Fourth, the mean map was obtained by voxel‐wise calculation of the random‐effects mean of the study maps.

Additionally, a jackknife sensitivity analysis was conducted to assess the reproducibility of the results and meta‐regression analyses with age, sex, and duration as regressors to help characterize heterogeneity with simple linear regression models. We did not analyze the average years of education, Hamilton Depression Scale (HAMD), or Young Manic Rating Scale (YMRS) because there are only three or four studies that have reported this information among all the included ones.

## 3 RESULT

### 3.1 Included studies

**FIGURE 1:**
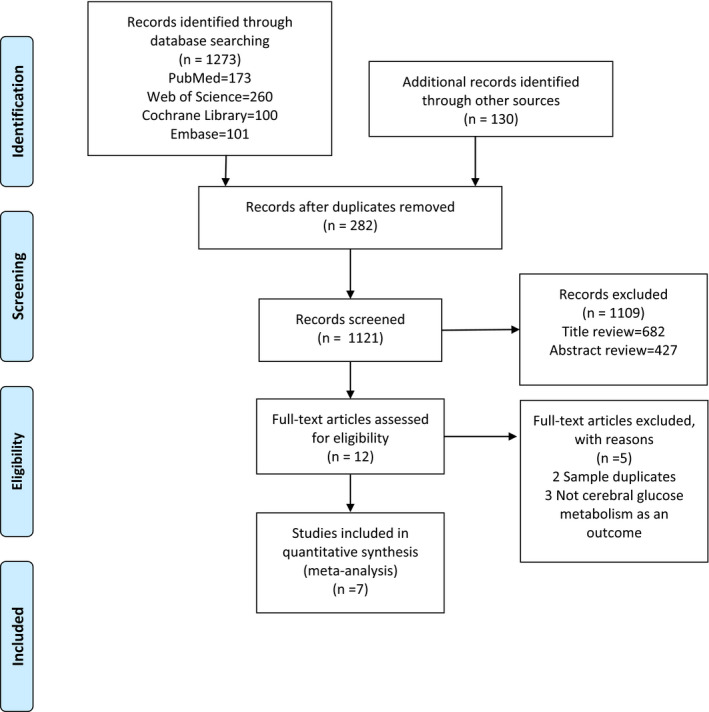
Flow diagram of studies identified, included, and excluded

**FIGURE 2:**
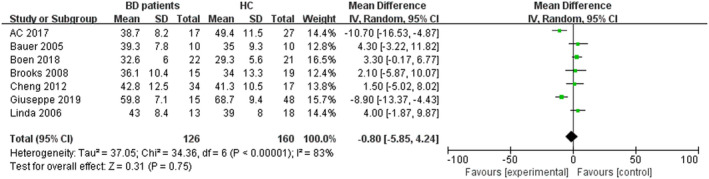
The stem‐and‐leaf plot of age difference between BD patients and healthy controls

**FIGURE 3:**
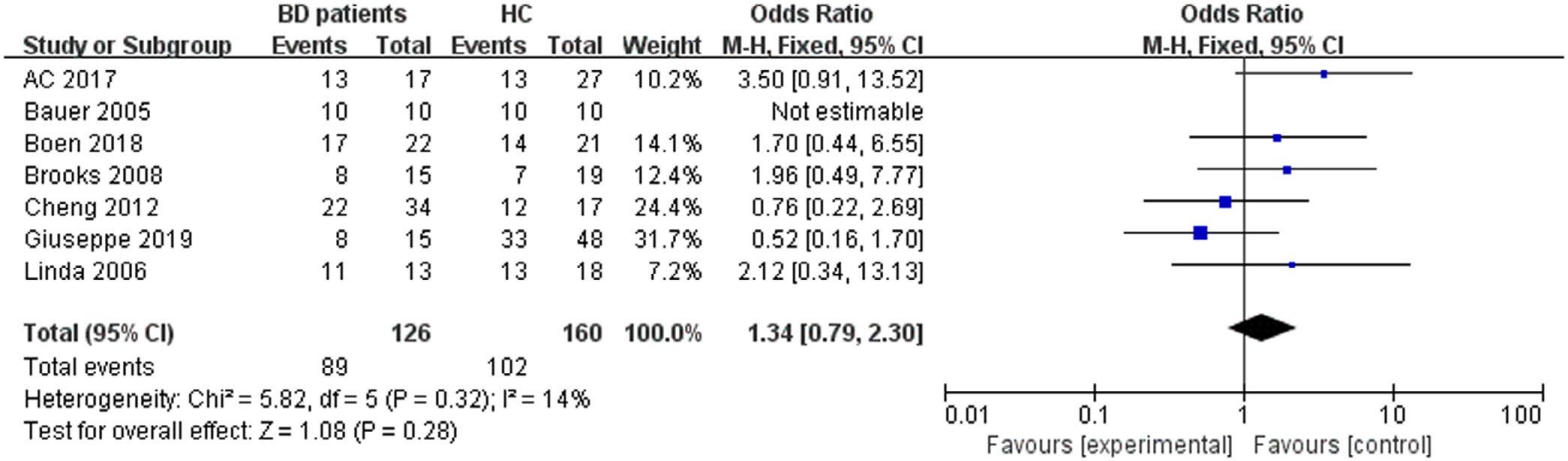
The stem‐and‐leaf plot of sex difference between BD patients and healthy controls

As showed in Figure 1, seven studies were included into this meta‐analysis comprising 126 individuals with BD and 160 healthy controls (Altamura et al., 2017; Bauer et al., 2005; Boen et al., 2018; Brooks et al., 2008; Delvecchio et al., 2019; Li et al., 2012; Mah et al., 2007). Among these studies, all patients met the DSM‐VI criteria for BD type I or II. Of these, two studies (Cheng Li et al. 2012; Delvecchio et al., 2019) included BD patients in the euthymic phase while scanning. The euthymic phase patients of Giuseppe's study met the standard that HAMD was lower than 10 points, while the YMRS was lower than 12 points. The study by Cheng et al defined a HAMD score of less than 9 points and YMRS of less than 7 points as euthymic phase. Three studies (Bauer et al., 2005; Brooks et al., 2008; Linda et al. 2007) included BD patients in depressive phase. Additionally, the study of Boen et al did not restrict the period in which the patients were included, while A.C. Altamura et al did not mention the stage in which the patients were enrolled. All 7 studies consisted of adult BD samples. The demographic and clinical characteristics of the participants are shown in Table 1, Figure 2, and Figure 3.

As shown in Table 1, among all 7 studies, 2 studies focused on BD II patients and 1 study was only focused on BD I patients. Giuseppe Delvecchio et al did not report the type of BD patients. Additionally, 3 other studies included both BD I patients and BD II patients, but only one of them reported the neuroimaging changes of BD I and BD II patients, respectively. Five of the studies showed the duration of the BD patients. The shortest duration among these 5 studies was 11.4 ± 7.0, and the longest was 22.9 ± 12.0.

We then used Revman 5.3 to analyze the differences in age and sex. The results are shown in Figure 2 and Figure 3. As the figures show, there were no significant differences in age (Z = 0.31, *p* =.75) and sex (Z = 1.08, *p* =.28) between BD patients and HC group. Among all the individuals in this meta‐analysis, the BD patient group included a total of 89 female patients and 37 male patients. In contrast, the HC group contained 102 female individuals and 58 males. The OR of sex between these two groups was 1.34 with a 95% confidence interval (95% Cl) of 0.79–2.30. The results of the analysis on age showed that the OR was −0.80 with a 95% Cl of −5.85–4.24.

### 3.2 Regional differences of brain glucose metabolism

**FIGURE 4:**
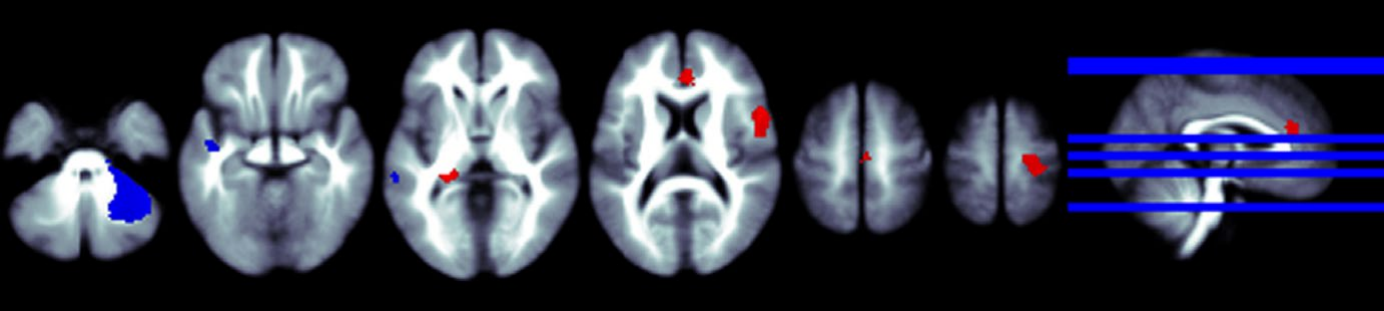
The regions that cerebral glucose metabolism change from BD patients to healthy control. *Slices are shown in axial view and marked with the z coordinate as distance in millimeters from the anterior–posterior commissure. The right side of the image corresponds to the right side of the brain. Higher cerebral glucose metabolism is indicated in red and lower cerebral glucose metabolism in blue

We obtained coordinates for the SDM analyses from all seven studies. As shown in Table 2 and Figure 4, patients with BD had significant cerebral glucose metabolism changes compared with healthy controls. The increases were mainly seen in the right precentral gyrus (BA 6), left anterior cingulate/paracingulate gyri (BA 24), and left optic radiations. Also, BD patients showed a significant decrease in bilateral brain glucose metabolism compared to the HC group in the left middle temporal gyrus (BA 21), left superior temporal gyrus (BA 48), and the middle cerebellar peduncles.

**TABLE 2:** Regional differences in cerebral glucose metabolism between individuals with BD and healthy controls

MNI coordinate	SDM‐Z	*p*	Voxels	Description
58,8,10	1.890	.000	262	Right rolandic operculum(BA 6)
2,36,14	1.783	.001	264	Left anterior cingulate / paracingulate gyri, BA 24
16,−20,72	1.653	.001	220	Right precentral gyrus, BA 6
−22,−32,2	1.837	.001	117	Left optic radiations
4,−18,56	1.645	.001	57	Right supplementary motor area, BA 6
18,−50,−32	−1.719	.000	2030	Middle cerebellar peduncles
−42,−14,−10	−1.535	.002	74	Left superior temporal gyrus, BA 48
−60,−32,−4	−1.522	.003	67	Left middle temporal gyrus, BA 21

Abbreviations: BA, Brodman's area; SDM, signed differential mapping.

### 3.3 Reliability

**FIGURE 5:**
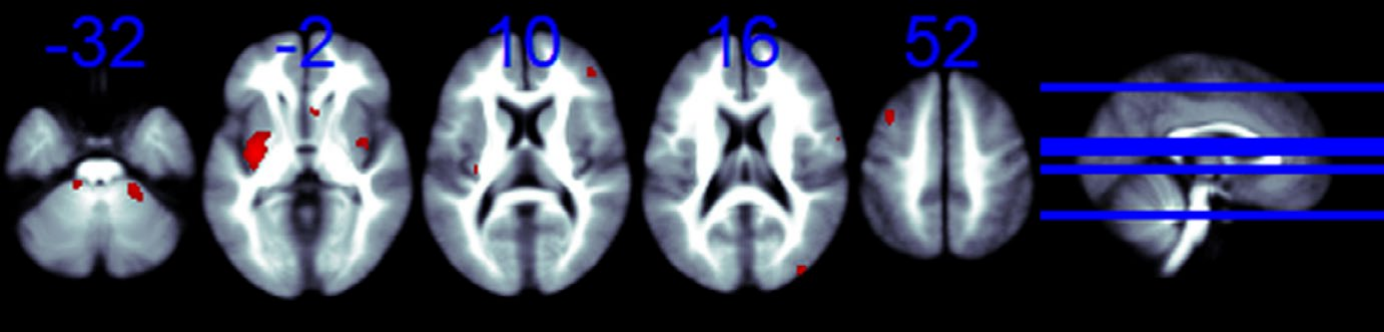
Heterogeneity analysis between BD patients and healthy controls. *Slices are shown in axial view and marked with the z coordinate as distance in millimeters from the anterior–posterior commissure. The right side of the image corresponds to the right side of the brain

To assess the reliability of this study, we analyzed the heterogeneity and sensitivity of this meta‐analysis by SDM. The heterogeneity analysis is shown in Figure 5. Jackknife sensitivity analyses revealed that the deficits in BA48 were highly robust, as it was replicable in all 7 studies. Differences in BA21, BA6, and cerebellum were highly replicable, as they remained significant in 5 studies. In BA24 and the left optic radiations, the differences are less replicable with only 4 studies remaining significant (Table 3).

**TABLE 3:** The Jackknife sensitivity analysis

Discarded study	BA48	BA21	BA6	cerebellum	BA24	Left optic radiations
Bauer (2005)	Yes	Yes	Yes	No	No	No
Linda_Mah, (2006)	Yes	Yes	Yes	Yes	Yes	No
Brooks, (2008)	Yes	No	Yes	Yes	Yes	Yes
Cheng, (2012)	Yes	Yes	Yes	Yes	No	Yes
A_C_Altamura, (2017)	Yes	No	No	Yes	No	No
Boen, (2018)	Yes	Yes	No	No	Yes	Yes
Giuseppe_Delvecchio, (2019)	Yes	Yes	Yes	Yes	Yes	Yes
Total	7 out of 7	5 out of 7	5 out of 7	5 out of 7	4 out of 7	4 out of 7

Abbreviation: BA, Brodmann area

### 3.4 Meta‐regression analysis

We conducted several regressors of moderating variables across studies, including sex distribution, mean age, and illness duration. Statistical thresholds at uncorrected *p* <.001 and cluster extent of 20 voxels were used (Table 4).

**TABLE 4:** Meta‐regression results showing an association between education/gender and cerebral glucose metabolism of BD patients

MNI coordinate	SDM‐Z	*p*	Voxels	Description
Effect of duration				
24,6,−6	2.495	.000	209	Right lenticular nucleus, putamen, BA48
−22,−32,−6	2.439	.000	43	Left optic radiations
8,34,20	−2.500	.000	295	Right anterior cingulate/paracingulate gyri
Effect of sex				
−20,60,16	2.767	.000	119	Left superior frontal gyrus, dorsolateral, BA10
42,34,26	2.376	.000	71	Right inferior frontal gyrus, triangular part, BA45
32,−2,−22	−3.350	.000	625	Right inferior network, inferior longitudinal fasciculus

The results of meta‐regression analysis on illness duration showed that a higher female ratio of BD patients was related to increased cerebral glucose metabolism in the right lenticular nucleus, putamen, BA48, and left optic radiations, as well as decreased cerebral glucose metabolism in the right anterior cingulate and paracingulate gyri. The results on sex distribution showed that a longer illness duration of BD patients was related to increased cerebral glucose metabolism in the right inferior frontal gyrus, triangular part, BA45, left superior frontal gyrus, dorsolateral, and BA10. The correlation between a longer illness duration and decreased cerebral glucose metabolism was shown in the right inferior network and inferior longitudinal fasciculus. According to our findings, there was no correlation between the mean age of BD patients and changes in cerebral glucose metabolism.

## 4 DISCUSSION

This meta‐analysis was aimed to investigate the reported characteristics of cerebral glucose metabolism in BD patients. To the best of our knowledge, our work is the first meta‐analysis of whole‐brain voxel‐based morphometry studies regarding the cerebral glucose metabolism in BD patients compared with healthy controls. Our main result showed that BD patients have higher cerebral glucose metabolism in certain brain regions including the right precentral gyrus (BA 6), left anterior cingulate/paracingulate gyri (BA 24), and left optic radiations; additionally, the metabolism was lower in the left middle temporal gyrus (BA 21), left superior temporal gyrus (BA48), and the middle cerebellar peduncles. Among all the regions, the most plausible result was the altered level of cerebral glucose metabolism in the left superior temporal (BA 48). We also found a relationship between cerebral glucose metabolism of BD patients and their sex and illness duration, which might be the source of heterogeneity between the literature.

Previous neuroimaging studies on BD have shown that there are clear functional and structural changes in neural circuits related to emotion, reward, and cognitive processing in patients (Fournier et al., ,,^2013^, ^2016^). Our study further supports this conclusion from the perspective of cerebral glucose metabolism.

The results of our study showed that there were significant alterations in cerebral glucose metabolism levels in the left superior temporal gyrus and middle temporal gyrus as well as the left cingulate and paracingulate gyrus in BD patients. This result is similar to the results of previous studies on bipolar disorder. A study by Delvecchio et al (Delvecchio et al., 2012) on functional MRI changes showed that bipolar disorder was associated with hyperactivity of the limbic system. The DTI study also found that the white matter of the limbic system, especially the cingulate gyrus, is affected by bipolar disorder (McKenna et al., 2015; Sarrazin et al., 2014). In addition, the findings of structural MRI have also revealed certain changes in the volume of the prefrontal lobe, the parahippocampal gyrus, and the cingulate cortex in BD patients (Lan et al., 2014; Redlich et al., 2014). Our study demonstrates that functional changes in the limbic system are common to BD patients at all stages of the disease. This reflects the changes in emotion management and cognitive function in patients with bipolar disorder, and suggests that the functional changes in the limbic system might be one of the disease characteristics of bipolar disorder and provide some reference value for the diagnosis of bipolar disorder.

The frontal lobe is the most advanced part of the brain, accounting for 40%–50% of the entire brain volume. The frontal lobe is closely related to human advanced neuropsychiatric activities, especially speech fluency, memory, and executive function (Whitfield‐Gabrieli & Ford, 2012). Among the Brodmann areas, BA 9, 10, and 11 mainly perform cognitive‐related functions and have a clear relationship with controlling emotions, dealing with problems, and processing and recalling information. The function of BA6 is closely related to sports, learning, planning, and working memory, and BA21 is responsible for auditory processing and language acceptance. Previous imaging studies have found abnormalities in the frontal lobe of patients with BD. A meta‐analysis suggested a significant reduction in the volume of the prefrontal lobe in patients with BD (Selvaraj et al., 2012). Some studies have also found that cerebral blood perfusion in the anterior frontal lobe of BD patients is significantly lower than normal (Alústiza et al., 2017; Arnone, 2009). Our results show that cerebral glucose metabolism in BA6 brain regions is significantly higher in BD patients than in normal controls, which may be closely related to the cognitive behavioral changes such as changes in learning ability and increased planning in BD patients.

Many studies have confirmed that there is a relationship between the cerebellum and advanced cognitive processing and emotional function (Shakiba, 2014). Schmahmann JD et al (Gomez‐Beldarrain & Garcia‐Monco, 1998) proposed cerebellar cognitive emotion syndrome, which includes executive dysfunction, spatial cognitive impairment, speech difficulties, and personality changes. Studies have suggested that these changes are associated with the regulation of damage in the cerebellar nerve circuit (Schmahmann et al., 1999). The results of a retrospective study also suggest that lesions in the cerebellar lobules VI, VII, VIII, etc. are associated with cognitive impairment (Parker et al., 2013). In another study, transcranial magnetic stimulation was performed on one side of the cerebellum of healthy volunteers, and cerebellar changes were detected by PET (Cho et al., 2012). The results show that the cranial magnetic stimulation of the cerebellum is directly related to changes in the excitability of the cognitive and emotion‐related brain regions, further demonstrating the role of the cerebellum in advanced cognitive function and emotion. An existing morphological study of patients with bipolar disorder found that the cerebellar crania volume and gray matter density of BD patients changed significantly compared with the HC group (Loeber, 1999), but the results are still controversial and call for further elucidation. Functional imaging studies have also suggested that cerebellar cerebral blood volume and cerebral blood flow in BD patients are different from HC groups, but the specific changes have differing results with the treatments and changes in mania and depression. Our results suggest that the level of brain glucose metabolism in patients with BD is lower than that of HC. Whether or not this change is related to the changes in emotion and cognition of BD patients requires further investigation.

In addition to acting as a processing center for auditory information, the temporal lobe is also an important brain region for the cognitive regulation of emotions (Clark, 2018). Previous studies have shown that patients with temporal lobe damage may show signs of depression, mania, and other mood disorders (de Oliveira et al., 2010; Salzberg et al., 2006). In addition, previous studies on depression have shown alterations in temporal lobe function in depressed patients (Ramezani et al., 2014). Considering that the temporal lobe is one of the major brain regions involved in seizures (Allone et al., 2017; Pascual, 2007), and previous studies have shown a clear role for antiepileptic drugs such as valproate in the treatment of bipolar disorder (Anderson et al., 2012; Müller & Leweke, 2016), does this mean that there are similar neurocirculatory changes in bipolar disorder and epilepsy? What are its possible changes? All of these need to be further explored.

The basal ganglia receive the evoked potential of the cerebral cortex and pass through the efferent projection fibers to form the basal ganglion circuit, which is then returned to the cerebral cortex by the thalamus (Graybiel, 2004). Through this neural circuit, the basal ganglia coordinate the regulation of the body, limbic system, and prefrontal function. Many studies have shown that, in addition to participating in autonomous movements, the basal ganglia play an important role in a variety of advanced cognitive functions such as thinking, language, emotion, memory, and learning. Previous studies have also suggested that significant changes in the volume of basal ganglia and cerebral blood perfusion are present in BD patients (Pompei et al., 2011; Toma et al., 2018). It is generally believed that cerebral blood perfusion is directly proportional to the level of glucose metabolism. However, many studies have suggested that cerebral blood perfusion in the lower cortex of the peripheral body is negatively correlated with the level of brain glucose metabolism (Devor et al., 2008), but more research is needed to prove whether or not there are similar characteristics in the brain metabolism of BD patients. However, our study did not find obvious differences in cerebral glucose metabolism in the basal ganglia.

There have been few reports on sex differences in neuroimaging in bipolar disorder patients. The results of our study showed that alterations in glucose metabolism in the inferior frontal gyrus in BD patients were sex‐dependent and are more pronounced in females than in males. A previous study of brain volume and structure in BD patients showed that the brain regions of male patients were larger than those of male controls, while the brain regions of female patients were smaller than those of healthy controls with the same sex (Mackay et al., 2010). Whether there is a correlation between this volume change and the level of glucose metabolism in the brain needs to be further confirmed by more gender‐specific studies. In addition, altered glucose metabolism in the right cingulate gyrus and medial temporal lobe in BD patients was significantly correlated with the duration of the disease. This suggests that altered cingulate glucose metabolism may be one of the potential indicators of disease duration and may be useful in determining the progression and outcome of bipolar disorder.

In conclusion, the results of this study showed that bipolar disorder mainly affects emotion management functions and cognitive functions. The alterations of cerebral glucose metabolism in the limbic network might be a new possible way for the identification of this disease. In addition, altered cerebral glucose metabolism levels were more pronounced in female patients and altered cingulate glucose metabolism may be one of the potential indicators of disease duration and may be useful in determining the progression and outcome of bipolar disorder.

### 4.1 Limitations and perspectives

Some limitations of the current study should be noted. First, although SDM is a new coordinate‐based meta‐analytic approach with strong power in identifying the convergence across neuroimaging studies, it was based on coordinate data from published results rather than original images. Second, some patients' HAMA, HAMD, YAMRS, and the information on the medication were incomplete, so it was impossible to judge the relationship between the severity of the disease and the treatment mode and the level of changes in cerebral glucose metabolism. Third, due to the incomplete description of disease staging in patients with bipolar disorder in the included literature, it was not possible to perform relevant analyses. Fourth, the study is based on clinical topics, so the mechanisms involved in the changes in cerebral glucose metabolism need further study. Fifth, this meta‐analysis was done at a study level and cannot address findings at the patient level. Finally, due to the current research on the changes of cerebral glucose metabolism in BD patients, some controversial results still need to be confirmed by more studies.

There have been many studies on cerebral blood flow, cerebral glucose metabolism, cerebral morphology, and other cerebral metabolic processes in BD patients, but there is currently no relevant research on the relationship between these factors. The pathogenesis of mental illness has always been a significant focus of research in the field, and whether or not these pathogeneses can be further elucidated through neuroimaging studies, which can further aim to provide new diagnostic and therapeutic ideas, is an area of psychiatry that remains to be discovered, calling for more studies.

## 5 CONCLUSION

Cerebral glucose metabolism alterations in the brain regions are likely to reflect the disease‐related functional abnormalities such as emotion and cognition. The most consistent and robust findings of this study were an increased cerebral glucose metabolism in the right precentral gyrus and the decrease in the left superior temporal gyrus, the left middle temporal gyrus, and cerebellum. Also, the gender distribution, mean age, and illness duration had significant moderating effects on cerebral glucose metabolism alterations. These findings contribute to a better understanding of the neurobiological underpinnings of bipolar disorder and may help to develop a new method in accurately diagnosing BD.

## CONFLICT OF INTEREST

We declare that we do not have any commercial or associative interest that represents a conflict of interest in connection with the work submitted.

## AUTHOR CONTRIBUTION

Chujun Wu and Chutong Ren designed this meta‐analysis, searched the literature, analyzed experimental results, and wrote the manuscript. Ziwei Teng and Sujuan Li researched the literature and decided whether the literatures were included when disagreement appeared. Floyd Silva embellished the language. Jindong Chen, Bolun Wang, and Haishan Wu guided, reviewed, and revised the manuscript and provided unique insights into the direction of the discussion.

## ETHICAL STATEMENT

This research is a meta‐analysis and does not require ethical statements.

## Data Availability

This research was a meta‐analysis, and all data used during the study appear in the published articles mentioned in this article.

## References

[brb32117-bib-0001] Aihara, M. , Ida, I. , Yuuki, N. , Oshima, A. , Kumano, H. , Takahashi, K. , Fukuda, M. , Oriuchi, N. , Endo, K. , Matsuda, H. , & Mikuni, M. (2007). HPA axis dysfunction in unmedicated major depressive disorder and its normalization by pharmacotherapy correlates with alteration of neural activity in prefrontal cortex and limbic/paralimbic regions. Psychiatry Research, 155(3), 245–256. 10.1016/j.pscychresns.2006.11.002 17587554

[brb32117-bib-0002] Allone, C. , Lo Buono, V. , Corallo, F. , Pisani, L. R. , Pollicino, P. , Bramanti, P. , & Marino, S. (2017). Neuroimaging and cognitive functions in temporal lobe epilepsy: A review of the literature. Journal of the Neurological Sciences, 381, 7–15. 10.1016/j.jns.2017.08.007 28991719

[brb32117-bib-0003] Altamura, A. C. , Delvecchio, G. , Marotta, G. , Oldani, L. , Pigoni, A. , Ciappolino, V. , Caletti, E. , Rovera, C. , Dobrea, C. , Arici, C. , Benatti, B. , Camuri, G. , Prunas, C. , Paoli, R. A. , Dell’osso, B. , Cinnante, C. , Triulzi, F. M. , & Brambilla, P. (2017). Structural and metabolic differentiation between bipolar disorder with psychosis and substance‐induced psychosis: An integrated MRI/PET study. Eur Psychiatry, 41, 85–94. 10.1016/j.eurpsy.2016.09.009 28049086

[brb32117-bib-0004] Alústiza, I. , Radua, J. , Pla, M. , Martin, R. , & Ortuño, F. (2017). Meta‐analysis of functional magnetic resonance imaging studies of timing and cognitive control in schizophrenia and bipolar disorder: Evidence of a primary time deficit. Schizophrenia Research, 188, 21–32. 10.1016/j.schres.2017.01.039 28169089

[brb32117-bib-0005] Anderson, I. M. , Haddad, P. M. , & Scott, J. (2012). Bipolar disorder. BMJ, 345, e8508. 10.1136/bmj.e8508 23271744

[brb32117-bib-0006] Arnone D. , Cavanagh J. , Gerber D. , Lawrie S. M. , Ebmeier K. P. , McIntosh A. M. (2009). Magnetic resonance imaging studies in bipolar disorder and schizophrenia: meta‐analysis. British Journal of Psychiatry, 195(3), 194–201. 10.1192/bjp.bp.108.059717 19721106

[brb32117-bib-0007] Bauer, M. , London, E. D. , Rasgon, N. , Berman, S. M. , Frye, M. A. , Altshuler, L. L. , Mandelkern, M. A. , Bramen, J. , Voytek, B. , Woods, R. , Mazziotta, J. C. , & Whybrow, P. C. (2005). Supraphysiological doses of levothyroxine alter regional cerebral metabolism and improve mood in bipolar depression. Molecular Psychiatry, 10(5), 456–469. 10.1038/sj.mp.4001647 15724143

[brb32117-bib-0008] Bhardwaj, R. , Chakrabarti, S. , Mittal, B. R. , & Sharan, P. (2010). A single photon emission computerized tomography (SPECT) study of regional cerebral blood flow in bipolar disorder. The World Journal of Biological Psychiatry, 11, 334–343. 10.3109/15622970802575977 20218796

[brb32117-bib-0009] Boen, E. , Hjørnevik, T. , Hummelen, B. , Elvsåshagen, T. , Moberget, T. , Holtedahl, J. E. , Babovic, A. , Hol, P. K. , Karterud, S. , & Malt, U. F. (2018). Patterns of altered regional brain glucose metabolism in borderline personality disorder and bipolar II disorder. Acta Psychiatrica Scand 139(3):256–268. 10.1111/acps.12997 30552759

[brb32117-bib-0010] Brody, A. L. , Saxena, S. , Stoessel, P. , Gillies, L. A. , Fairbanks, L. A. , Alborzian, S. , Phelps, M. E. , Huang, S.‐C. , Wu, H.‐M. , Ho, M. L. , Ho, M. K. , Au, S. C. , Maidment, K. , & Baxter, L. R. (2001). Regional brain metabolic changes in patients with major depression treated with either paroxetine or interpersonal therapy: Preliminary findings. Archives of General Psychiatry, 58(7), 631–640.– 10.1001/archpsyc.58.7.631 11448368

[brb32117-bib-0011] Brooks, J. O. III , & Vizueta, N. (2014). Diagnostic and clinical implications of functional neuroimaging in bipolar disorder. Journal of Psychiatric Research, 57, 12–25. 10.1016/j.jpsychires.2014.05.018 25015683

[brb32117-bib-0012] Brooks, J. O. , Wang, P. W. , Bonner, J. C. , Rosen, A. C. , Hoblyn, J. C. , Hill, S. J. , & Ketter, T. A. (2008). Decreased prefrontal, anterior cingulate, insula, and ventral striatal metabolism in medication‐free depressed outpatients with bipolar disorder. Journal of Psychiatric Research, 43(3), 181–188. 10.1016/j.jpsychires.2008.04.015 18582900 PMC3265392

[brb32117-bib-0013] Cho, S. S. , Yoon, E. J. , Bang, S. A. , Park, H. S. , Kim, Y. K. , Strafella, A. P. , & Kim, S. E. (2012). Metabolic changes of cerebrum by repetitive transcranial magnetic stimulation over lateral cerebellum: A study with FDG PET. Cerebellum, 11(3), 739–748. 10.1007/s12311-011-0333-7 22161500

[brb32117-bib-0014] Clark, R. E. (2018). Current topics regarding the function of the medial temporal lobe memory system. Current Topics in Behavioral Neurosciences, 37, 13–42. 29589322 10.1007/7854_2017_481

[brb32117-bib-0015] Davison, C. M. , & O'Brien, J. T. (2014). A comparison of FDG‐PET and blood flow SPECT in the diagnosis of neurodegenerative dementias: A systematic review. International Journal of Geriatric Psychiatry, 29(6), 551–561. 10.1002/gps.4036 24123413

[brb32117-bib-0016] de Oliveira, G. N. M. , Kummer, A. , Salgado, J. V. , Portela, E. J. , Sousa‐Pereira, S. R. , David, A. S. , & Teixeira, A. L. (2010). Psychiatric disorders in temporal lobe epilepsy: An overview from a tertiary service in Brazil. Seizure, 19(8), 479–484. 10.1016/j.seizure.2010.07.004 20708951

[brb32117-bib-0017] Delvecchio, G. , Fossati, P. , Boyer, P. , Brambilla, P. , Falkai, P. , Gruber, O. , Hietala, J. , Lawrie, S. M. , Martinot, J.‐L. , McIntosh, A. M. , Meisenzahl, E. , & Frangou, S. (2012). Common and distinct neural correlates of emotional processing in Bipolar Disorder and Major Depressive Disorder: A voxel‐based meta‐analysis of functional magnetic resonance imaging studies. European Neuropsychopharmacology, 22(2), 100–113. 10.1016/j.euroneuro.2011.07.003 21820878

[brb32117-bib-0018] Delvecchio, G. , Mandolini, G. M. , Arighi, A. , Prunas, C. , Mauri, C. M. , Pietroboni, A. M. , Marotta, G. , Cinnante, C. M. , Triulzi, F. M. , Galimberti, D. , Scarpini, E. , Altamura, A. C. , & Brambilla, P. (2019). Structural and metabolic cerebral alterations between elderly bipolar disorder and behavioural variant frontotemporal dementia: A combined MRI‐PET study. Australian and New Zealand Journal of Psychiatry, 53(5), 413–423. 10.1177/0004867418815976 30545239

[brb32117-bib-0019] Devor, A. , Hillman, E. M. C. , Tian, P. , Waeber, C. , Teng, I. C. , Ruvinskaya, L. , Shalinsky, M. H. , Zhu, H. , Haslinger, R. H. , Narayanan, S. N. , Ulbert, I. , Dunn, A. K. , Lo, E. H. , Rosen, B. R. , Dale, A. M. , Kleinfeld, D. , & Boas, D. A. (2008). Stimulus‐induced changes in blood flow and 2‐deoxyglucose uptake dissociate in ipsilateral somatosensory cortex. Journal of Neuroscience, 28(53), 14347–14357. 10.1523/JNEUROSCI.4307-08.2008 19118167 PMC2655308

[brb32117-bib-0020] Fournier, J. C. , Chase, H. W. , Almeida, J. , & Phillips, M. L. (2016). Within‐ and between‐session changes in neural activity during emotion processing in unipolar and bipolar depression. Biol Psychiatry Cogn Neurosci Neuroimaging, 1(6), 518–527. 10.1016/j.bpsc.2016.03.005 28083566 PMC5220672

[brb32117-bib-0021] Fournier, J. C. , Keener, M. T. , Almeida, J. , Kronhaus, D. M. , & Phillips, M. L. (2013). Amygdala and whole‐brain activity to emotional faces distinguishes major depressive disorder and bipolar disorder. Bipolar Disorders, 15(7), 741–752. 10.1111/bdi.12106 23911154 PMC3864629

[brb32117-bib-0022] Gomez‐Beldarrain, M. , & Garcia‐Monco, J. C. (1998). The cerebellar cognitive affective syndrome. Brain, 121(Pt 11), 2202–2205. 10.1093/brain/121.11.2202 9827779

[brb32117-bib-0023] Grande, I. , Berk, M. , Birmaher, B. , & Vieta, E. (2016). Bipolar disorder. Lancet, 387(10027), 1561–1572. 10.1016/S0140-6736(15)00241-X 26388529

[brb32117-bib-0024] Graybiel, A. M. (2004). Network‐level neuroplasticity in cortico‐basal ganglia pathways. Parkinsonism & Related Disorders, 10(5), 293–296. 10.1016/j.parkreldis.2004.03.007 15196508

[brb32117-bib-0025] Huang, Y. , Wang, Y. U. , Wang, H. , Liu, Z. , Yu, X. , Yan, J. , Yu, Y. , Kou, C. , Xu, X. , Lu, J. , Wang, Z. , He, S. , Xu, Y. , He, Y. , Li, T. , Guo, W. , Tian, H. , Xu, G. , Xu, X. , … Wu, Y. (2019). Prevalence of mental disorders in China: A cross‐sectional epidemiological study. Lancet Psychiatry, 6(3), 211–224. 10.1016/S2215-0366(18)30511-X 30792114

[brb32117-bib-0026] Ido, T. , Wan, C.‐N. , Casella, V. , Fowler, J. S. , Wolf, A. P. , Reivich, M. , & Kuhl, D. E. (1978). Labeled 2‐deoxy‐D‐glucose analogs: 18F‐labeled 2‐deoxy‐2‐fluoro‐D‐glucose, 2‐deoxy‐2‐fluoro‐D‐mannose and 14C‐2‐deoxy‐2‐fluoro‐D‐glucose. Journal of Labelled Compounds and Radiopharmaceuticals, 14, 175–183. 10.1002/jlcr.2580140204

[brb32117-bib-0027] Kegeles, L. S. , Malone, K. M. , Slifstein, M. , Ellis, S. P. , Xanthopoulos, E. , Keilp, J. G. , Campbell, C. , Oquendo, M. , Van Heertum, R. L. , & Mann, J. J. (2003). Response of cortical metabolic deficits to serotonergic challenge in familial mood disorders. American Journal of Psychiatry, 160(1), 76–82. 10.1176/appi.ajp.160.1.76 12505804

[brb32117-bib-0028] Kennedy, S. H. , Evans, K. R. , Krüger, S. , Mayberg, H. S. , Meyer, J. H. , McCann, S. , Arifuzzman, A. I. , Houle, S. , & Vaccarino, F. J. (2001). Changes in regional brain glucose metabolism measured with positron emission tomography after paroxetine treatment of major depression. American Journal of Psychiatry, 158(6), 899–905. 10.1176/appi.ajp.158.6.899 11384897

[brb32117-bib-0029] Kessler, R. C. , Chiu, W. T. , Demler, O. , & Walters, E. E. (2005). Prevalence, severity, and comorbidity of 12‐month DSM‐IV disorders in the National Comorbidity Survey Replication. Archives of General Psychiatry, 62(6), 617–627.– 10.1001/archpsyc.62.6.617 15939839 PMC2847357

[brb32117-bib-0030] Lan, M. J. , Chhetry, B. T. , Oquendo, M. A. , Sublette, M. E. , Sullivan, G. , Mann, J. J. , & Parsey, R. V. (2014). Cortical thickness differences between bipolar depression and major depressive disorder. Bipolar Disorders, 16(4), 378–388. 10.1111/bdi.12175 24428430 PMC4047134

[brb32117-bib-0031] Li, C.‐T. , Hsieh, J.‐C. , Wang, S.‐J. , Yang, B.‐H. , Bai, Y.‐M. , Lin, W.‐C. , Lan, C.‐C. , & Su, T.‐P. (2012). Differential relations between fronto‐limbic metabolism and executive function in patients with remitted bipolar I and bipolar II disorder. Bipolar Disorders, 14(8), 831–842. 10.1111/bdi.12017 23167933

[brb32117-bib-0032] Liberati, A. , Altman, D. G. , Tetzlaff, J. , Mulrow, C. , Gotzsche, P. C. , Ioannidis, J. P. A. , Clarke, M. , Devereaux, P. J. , Kleijnen, J. , & Moher, D. (2009). The PRISMA statement for reporting systematic reviews and meta‐analyses of studies that evaluate healthcare interventions: Explanation and elaboration. BMJ, 339, b2700. 10.1136/bmj.b2700 19622552 PMC2714672

[brb32117-bib-0033] Lim, L. , Radua, J. , & Rubia, K. (2014). Gray matter abnormalities in childhood maltreatment: A voxel‐wise meta‐analysis. American Journal of Psychiatry, 171(8), 854–863. 10.1176/appi.ajp.2014.13101427 24781447

[brb32117-bib-0034] Loeber, R. T. et al (1999). Differences in cerebellar blood volume in schizophrenia and bipolar disorder. Schizophrenia Research, 37(1), 81–89. 10.1016/S0920-9964(98)00137-6 10227110

[brb32117-bib-0035] Mackay, C. E. , Roddick, E. , Barrick, T. R. , Lloyd, A. J. , Roberts, N. , Crow, T. J. , Young, A. H. , & Ferrier, I. N. (2010). Sex dependence of brain size and shape in bipolar disorder: An exploratory study. Bipolar Disorders, 12(3), 306–311. 10.1111/j.1399-5618.2010.00804.x 20565437

[brb32117-bib-0036] Maggioni, E. , Altamura, A. C. , & Brambilla, P. (2017). Exploring the neuroanatomical bases of psychotic features in bipolar disorder. Epidemiol Psychiatr Sci, 26(4), 358–363. 10.1017/S2045796017000087 28343462 PMC6998627

[brb32117-bib-0037] Mah, L. , Zarate, C. A. , Singh, J. , Duan, Y.‐F. , Luckenbaugh, D. A. , Manji, H. K. , & Drevets, W. C. (2007). Regional cerebral glucose metabolic abnormalities in bipolar II depression. Biological Psychiatry, 61(6), 765–775. 10.1016/j.biopsych.2006.06.009 17027930

[brb32117-bib-0038] McKenna, B. S. , Theilmann, R. J. , Sutherland, A. N. , & Eyler, L. T. (2015). Fusing functional MRI and diffusion tensor imaging measures of brain function and structure to predict working memory and processing speed performance among inter‐episode bipolar patients. Journal of the International Neuropsychological Society, 21(5), 330–341. 10.1017/S1355617715000314 26037664 PMC4655813

[brb32117-bib-0039] Mergenthaler, P. , Lindauer, U. , Dienel, G. A. , & Meisel, A. (2013). Sugar for the brain: The role of glucose in physiological and pathological brain function. Trends in Neurosciences, 36(10), 587–597. 10.1016/j.tins.2013.07.001 23968694 PMC3900881

[brb32117-bib-0040] Müller, J. K. , & Leweke, F. M. (2016). Bipolar disorder: Clinical overview. Medizinische Monatsschrift Fur Pharmazeuten, 39(9), 363–369. 29956510

[brb32117-bib-0041] Murray, C. J. L. , Vos, T. , Lozano, R. , Naghavi, M. , Flaxman, A. D. , Michaud, C. , Ezzati, M. , Shibuya, K. , Salomon, J. A. , Abdalla, S. , Aboyans, V. , Abraham, J. , Ackerman, I. , Aggarwal, R. , Ahn, S. Y. , Ali, M. K. , AlMazroa, M. A. , Alvarado, M. , Anderson, H. R. , … Lopez, A. D. (2012). Disability‐adjusted life years (DALYs) for 291 diseases and injuries in 21 regions, 1990–2010: A systematic analysis for the Global Burden of Disease Study 2010. Lancet, 380(9859), 2197–2223. 10.1016/S0140-6736(12)61689-4 23245608

[brb32117-bib-0042] Parker, K. L. , Andreasen, N. C. , Liu, D. , Freeman, J. H. , & O'Leary, D. S. (2013). Eyeblink conditioning in unmedicated schizophrenia patients: A positron emission tomography study. Psychiatry Research, 214(3), 402–409. 10.1016/j.pscychresns.2013.07.006 24090512 PMC3980571

[brb32117-bib-0043] Pascual, M. R. (2007). Temporal lobe epilepsy: Clinical semiology and neurophysiological studies. Seminars in Ultrasound, CT and MR, 28(6), 416–423. 10.1053/j.sult.2007.09.004 18074998

[brb32117-bib-0044] Patlak, C. S. , Blasberg, R. G. , & Fenstermacher, J. D. (1983). Graphical evaluation of blood‐to‐brain transfer constants from multiple‐time uptake data. Journal of Cerebral Blood Flow and Metabolism, 3(1), 1–7. 10.1038/jcbfm.1983.1 6822610

[brb32117-bib-0045] Pompei, F. , Dima, D. , Rubia, K. , Kumari, V. , & Frangou, S. (2011). Dissociable functional connectivity changes during the Stroop task relating to risk, resilience and disease expression in bipolar disorder. NeuroImage, 57(2), 576–582. 10.1016/j.neuroimage.2011.04.055 21570470

[brb32117-bib-0046] Radua Joaquim , Rubia Katya , Canales‐Rodríguez Erick Jorge , Pomarol‐Clotet Edith , Fusar‐Poli Paolo , Mataix‐Cols David (2014). Anisotropic Kernels for Coordinate‐Based Meta‐Analyses of Neuroimaging Studies. Frontiers in Psychiatry, 5, 10.3389/fpsyt.2014.00013. PMC3919071 24575054

[brb32117-bib-0047] Radua, J. , Mataix‐Cols, D. , Phillips, M. L. , El‐Hage, W. , Kronhaus, D. M. , Cardoner, N. , & Surguladze, S. (2012). A new meta‐analytic method for neuroimaging studies that combines reported peak coordinates and statistical parametric maps. European Psychiatry, 27(8), 605–611. 10.1016/j.eurpsy.2011.04.001 21658917

[brb32117-bib-0048] Ramezani, M. , Johnsrude, I. , Rasoulian, A. , Bosma, R. , Tong, R. , Hollenstein, T. , Harkness, K. , & Abolmaesumi, P. (2014). Temporal‐lobe morphology differs between healthy adolescents and those with early‐onset of depression. NeuroImage Clinical, 6, 145–155. 10.1016/j.nicl.2014.08.007 25379426 PMC4215529

[brb32117-bib-0049] Redlich, R. , Almeida, J. J. R. , Grotegerd, D. , Opel, N. , Kugel, H. , Heindel, W. , Arolt, V. , Phillips, M. L. , & Dannlowski, U. (2014). Brain morphometric biomarkers distinguishing unipolar and bipolar depression. A voxel‐based morphometry‐pattern classification approach. JAMA Psychiatry, 71(11), 1222–1230.– 10.1001/jamapsychiatry.2014.1100 25188810 PMC5538312

[brb32117-bib-0050] Roalf, D. R. , & Gur, R. C. (2017). Functional brain imaging in neuropsychology over the past 25 years. Neuropsychology, 31(8), 954–971. 10.1037/neu0000426 29376672 PMC5822440

[brb32117-bib-0051] Salzberg, M. , Taher, T. , Davie, M. , Carne, R. , Hicks, R. J. , Cook, M. , Murphy, M. , Vinton, A. , & O'Brien, T. J. (2006). Depression in temporal lobe epilepsy surgery patients: An FDG‐PET study. Epilepsia, 47(12), 2125–2130. 10.1111/j.1528-1167.2006.00860.x 17201712

[brb32117-bib-0052] Sarrazin, S. , Poupon, C. , Linke, J. , Wessa, M. , Phillips, M. , Delavest, M. , Versace, A. , Almeida, J. , Guevara, P. , Duclap, D. , Duchesnay, E. , Mangin, J.‐F. , Le Dudal, K. , Daban, C. , Hamdani, N. , D’Albis, M.‐A. , Leboyer, M. , & Houenou, J. (2014). A multicenter tractography study of deep white matter tracts in bipolar I disorder: Psychotic features and interhemispheric disconnectivity. JAMA Psychiatry, 71(4), 388–396.– 10.1001/jamapsychiatry.2013.4513 24522197

[brb32117-bib-0053] Saxena, S. , Brody, A. L. , Ho, M. L. , Alborzian, S. , Ho, M. K. , Maidment, K. M. , Huang, S.‐C. , Wu, H.‐M. , Au, S. C. , & Baxter, L. R. (2001). Cerebral metabolism in major depression and obsessive‐compulsive disorder occurring separately and concurrently. Biological Psychiatry, 50(3), 159–170. 10.1016/S0006-3223(01)01123-4 11513814

[brb32117-bib-0054] Schmahmann, J. D. , Doyon, J. , McDonald, D. , Holmes, C. , Lavoie, K. , Hurwitz, A. S. , Kabani, N. , Toga, A. , Evans, A. , & Petrides, M. (1999). Three‐dimensional MRI atlas of the human cerebellum in proportional stereotaxic space. NeuroImage, 10(3 Pt 1), 233–260. 10.1006/nimg.1999.0459 10458940

[brb32117-bib-0055] Scholl, M. , Damian, A. , & Engler, H. (2014). Fluorodeoxyglucose PET in neurology and psychiatry. PET Clin, 9(4), pp. 371–90, v. 10.1016/j.cpet.2014.07.005 26050943

[brb32117-bib-0056] Selvaraj, S. , Arnone, D. , Job, D. , Stanfield, A. , Farrow, T. F. D. , Nugent, A. C. , Scherk, H. , Gruber, O. , Chen, X. , Sachdev, P. S. , Dickstein, D. P. , Malhi, G. S. , Ha, T. H. , Ha, K. , Phillips, M. L. , & McIntosh, A. M. (2012). Grey matter differences in bipolar disorder: A meta‐analysis of voxel‐based morphometry studies. Bipolar Disorders, 14(2), 135–145. 10.1111/j.1399-5618.2012.01000.x 22420589

[brb32117-bib-0057] Shakiba, A. (2014). The role of the cerebellum in neurobiology of psychiatric disorders. Neurologic Clinics, 32(4), 1105–1115. 10.1016/j.ncl.2014.07.008 25439296

[brb32117-bib-0058] Toma, S. , MacIntosh, B. J. , Swardfager, W. , & Goldstein, B. I. (2018). Cerebral blood flow in bipolar disorder: A systematic review. Journal of Affective Disorders, 241, 505–513. 10.1016/j.jad.2018.08.040 30149339

[brb32117-bib-0059] Tondo, L. , Vázquez, G. H. , & Baldessarini, R. J. (2017). Depression and mania in bipolar disorder. Current Neuropharmacology, 15(3), 353–358. 10.2174/1570159X14666160606210811 28503106 PMC5405618

[brb32117-bib-0060] Whitfield‐Gabrieli, S. , & Ford, J. M. (2012). Default mode network activity and connectivity in psychopathology. Annual Review of Clinical Psychology, 8, 49–76. 10.1146/annurev-clinpsy-032511-143049 22224834

